# “Notification! You May Have Cancer.” Could Smartphones and Wearables Help Detect Cancer Early?

**DOI:** 10.2196/52577

**Published:** 2024-05-20

**Authors:** Suzanne E Scott, Matthew J Thompson

**Affiliations:** 1 Centre for Cancer Screening, Prevention and Early Diagnosis Wolfson Institute of Population Health Queen Mary University of London London United Kingdom; 2 Department of Family Medicine University of Washington Seattle, WA United States

**Keywords:** wearables, early diagnosis, cancer, challenges, diagnosis, wearable, detect, detection, smartphone, cancer diagnosis, symptoms, monitoring, monitor, implementation, anxiety, health care service, mobile phone

## Abstract

This viewpoint paper considers the authors’ perspectives on the potential role of smartphones, wearables, and other technologies in the diagnosis of cancer. We believe that these technologies could be valuable additions in the pursuit of early cancer diagnosis, as they offer solutions to the timely detection of signals or symptoms and monitoring of subtle changes in behavior that may otherwise be missed. In addition to signal detection, technologies could assist symptom interpretation and guide and facilitate access to health care. This paper aims to provide an overview of the scientific rationale as to why these technologies could be valuable for early cancer detection, as well as outline the next steps for research and development to drive investigation into the potential for smartphones and wearables in this context and optimize implementation. We draw attention to potential barriers to successful implementation, including the difficulty of the development of signals and sensors with sufficient utility and accuracy through robust research with the target group. There are regulatory challenges; the potential for innovations to exacerbate inequalities; and questions surrounding acceptability, uptake, and correct use by the intended target group and health care practitioners. Finally, there is potential for unintended consequences on individuals and health care services including unnecessary anxiety, increased symptom burden, overinvestigation, and inappropriate use of health care resources.

## Introduction

There is growing use of smartphones, wearables, and other technologies in health and wellness, either as consumer products or medical devices. The *National Health Service (NHS) Long Term Plan* [1] anticipates that in 10 years, people will have “the option for their physiology to be effortlessly monitored by wearable devices. People will be helped to stay well, to recognize important symptoms early, and to manage their own health, guided by digital tools*.*” Similarly, in 2020, the US Food and Drug Administration (FDA) launched a *Digital Health Innovation Action Plan* to encourage digital health innovation as “digital health technologies can empower consumers to make better-informed decisions about their own health and provide new options for facilitating prevention, early diagnosis of life-threatening diseases, and management of chronic conditions outside of traditional care settings” [2]. Wearables are devices that can be worn to detect and monitor biometric data such as heart rate, blood oxygen saturation, sleep pattern, or temperature while the wearer continues their normal routines. A further category of wearables involves skin patches used to measure biochemical signals (ie, glucose) on a continuous basis that are increasingly being considered as a standard of care for individuals with certain conditions (eg, diabetes) [3]. While most wearables have been wrist-worn devices, similar physiologic signals are now being generated from other devices such as rings or earbuds. Smartphones are also increasingly being used for health and wellness; they have the advantage of far higher use (compared with wearables), and a growing array of different sensors are routinely embedded. Could smartphones and wearables help detect cancer and, importantly, detect cancer earlier in its disease course when it is more likely to be localized and with a better prognosis? This paper provides an overview of the scientific rationale as to why these technologies could be valuable for early detection of cancer, the potential barriers to successful implementation, and the next steps for research and development.

## Potential of Smartphones and Wearables for Early Detection of Cancer

While national cancer screening programs offer the opportunity to detect cancer or precancerous lesions in asymptomatic individuals, routine screening currently only accounts for the minority (<10%) of cancer diagnoses [4,5]. The predominant route to a cancer diagnosis is symptomatic presentation to ambulatory care. Thus, the diagnosis of cancer heavily relies on patients’ ability to notice and attend to relevant bodily changes and their decision to consult a health care professional [6,7]. However, noticing relevant bodily changes is challenging given the multiple subtle changes that may signal cancer among the plethora of daily bodily changes; fluctuations of normal bodily processes; self-limiting, transient symptoms; and the presence of chronic conditions. The signal-to-noise ratio is weak. This issue is exacerbated by individuals’ limited ability to accurately interpret vague bodily changes, many of which can be associated with cancer (eg, fatigue, weight loss, and stomach upset). This is because our awareness, attention, and interpretation are affected by expectations; emotions; beliefs; and biological, environmental, sociodemographic, and contextual factors [8-11]. Furthermore, symptoms may evolve very slowly over time, making it difficult to notice subtle changes. It is reported that the predominant risk factor for delay in seeking help following the detection of cancer symptoms is the “lack of interpretation by patients of the serious nature of their symptoms” [12].

Smartphones and wearable technologies have the potential to facilitate the detection and tracking of bodily changes that might otherwise be dismissed or interpreted as only needing self-medication rather than the attention of a health care professional. There is emerging data about early, subtle signs of cancer, and some of these may be amenable to detection by electronic sensors and monitoring of behavior (see Table 1).

**Table 1:** Potential signals of cancer that can be measured using sensors in smartphones or wearables.

Sensors currently available on some smartphones or wearables	Examples of signals for health features that could be related to cancer
Audio signals from microphones [13]	Changes in cough and breathing difficulty (associated with lung cancer) [14] Changes in voice such as hoarseness (associated with head and neck cancer and lung cancer) [14]
GPS location tracking and activity tracking [15]	Reduced activity resulting from fatigue (associated with multiple cancers) [14]
Image capture and analysis [16-18]	Anemia detected from images of the skin or eyes (associated with multiple cancers) [19] Jaundice detected from images of the skin or eyes (associated with pancreatic cancer) [20] Changes in skin lesions (associated with skin cancer) [14]
Temperature measurement [21]	Rise in temperature (associated with pancreatic cancer) [22]
Body composition using image analysis and electro dermal activity [23]	Weight loss (associated with multiple cancers) [14]
Photoplethysmogram [24]	Anemia (associated with multiple cancers) [19]

Sensors could allow the detection of changes prior to them being noticed or interpreted as symptoms, for example, a reduction in activity prior to fatigue or changes in food consumption prior to weight loss. There is recent evidence that monitoring day-to-day purchases could detect an increase in over-the-counter pain and indigestion medication 8 months prior to ovarian cancer diagnosis [25]. This demonstrates how tracking and monitoring change over time could allow insight into emerging disease. This is particularly useful for clinicians working in health care settings with limited time and resources and where cancer is a relatively rare occurrence among the burden of other diseases. In addition to the detection of signals, smartphones and wearables could alert the user to the need for health care consultation and provide an endorsement to seek care. This could overcome the commonly reported barrier to presentation (“concern about bothering the doctor”) that arises when there is uncertainty about the need for care [26-28].

## Potential Barriers to Successful Implementation

### Overview

Despite the promise of smartphones and wearables for early detection of cancer, there are several hurdles to implementation that require attention. Key barriers to success are outlined here, alongside suggestions for how these may be addressed with future research.

### Signals and Sensors With Accuracy and Utility

The use and adoption of smartphones and wearables in this context require robust research into the selection of a signal, the development of sensors, and the generating evidence of accuracy and utility of those sensors in the real world. This includes identifying physiologic (or emerging pathophysiologic) signals that are most predictive of cancer, determining how often these need to be collected, and elucidating what other data would add precision to results (eg, age, risk factors, and presence of symptoms). For technology developers, sensors are usually designed and prioritized for a number of potential applications, mainly targeting overall health and wellness rather than diagnostic capabilities per se. Prioritizing these research and development efforts for cancer detection specifically over and above other priorities could be challenging to justify for business development reasons. Relatedly, the original intended commercial purpose of existing sensors may not have been connected to cancer detection. To make headway in this field of research, technology developers and device users will need to be willing to provide access to data for research. General Data Protection Regulation allows device users to share their data with third-party organizations under the right to portability. Developing systems to facilitate data sharing, in formats compatible with health data, could allow the generation of new data sets to signal cancer risk. This will prevent duplication of effort and maximize the use of existing data for public benefit.

While initial evidence on the accuracy of a sensor to detect a given health signal could involve case-control studies (eg, individuals recently diagnosed with cancer and matched controls), subsequent research would likely require large prospective cohorts. Further, given the weak signal-to-noise ratio, it is likely that signals from wearables or smartphones alone might lack sensitivity or specificity. Therefore, research that combines signals from wearables or smartphones with other digital sources of data (eg, symptoms recorded in health records and initial laboratory tests in primary care) will almost certainly be needed to demonstrate sufficient accuracy and utility in target populations. This was recently highlighted in a systematic review [29] of artificial intelligence technologies for skin cancer detection. Despite an abundance of digital products, the review highlighted that there has been very little testing in low-prevalence populations and limited data on the use of lower-quality images (eg, taken by patients or family physicians or using lower-quality phones), and as such, widespread adoption into practice has been limited [30].

Innovators also need to consider (and test) whether these new digital tools should and could detect more than 1 type of cancer (or detect other potentially important nonmalignant diseases; eg, cirrhosis in individuals with jaundice or depression in individuals with weight loss). Other considerations include who the target group is (eg, all adults or only those at higher risk of developing cancer), at what point in time (eg, certain age), and at what periodicity that group should begin using this technology for the detection of cancer. Further, it is well documented that symptom monitoring increases selective attention to the body, resulting in increased symptom reporting [31]; thus, the monitoring of symptoms could result in increased symptom burden. Development and testing need to determine the extent to which the monitoring of activity, symptoms, and other signals changes the outputs of those measurements [32,33].

### Regulatory Challenges

Given the burden involved in fulfilling regulatory approvals for diagnostic devices, many smartphone technologies and wearables that could potentially have value for cancer detection will instead be introduced as products for overall health and wellness management. As the field expands, more guidance and standards for digital health tools are being introduced to ensure that they are not only safe and effective but also adoptable by the health care system [34]. For example, the recent UK National Institute of Health and Care Excellence (NICE) evidence standards framework [35] is intended to ensure new digital health technologies are clinically effective and offer value to the health care system. The framework includes standards concerning safety, quality, acceptability, bias mitigation, data practices, professional oversight, credibility with health professionals, safeguarding assurances, scalability, as well as evidence of real-world performance and use. In some countries, consumer protection regulations also determine standards that certain wellness features (eg, step counting and heart rate measurement) need to fulfill, even though these are not regulated medical devices. As specified in the CanTest framework for early cancer detection [36], research and development will benefit from this early specification of the criteria (eg, target product profiles) needed for successful digital products for cancer detection [37-39].

### Ensuring Equity

A key issue of wearables and smartphone technologies is the potential for new innovations to exacerbate inequalities in cancer outcomes. Sociodemographic factors such as household income, age, level of education, and gender have been found to influence the use of mobile health (mHealth) technology [40-44] and there is “a real risk that the increased use of digital technologies will make care experiences and outcomes worse for some people (or communities)” [45]. Development of wearables and smartphone technologies for cancer detection should be conducted with an equity lens to focus on the views and needs of those living or working in more deprived areas and those at risk of lower health literacy (eg, those with lower educational level, older age, lower income, and ethnic minority groups) [46,47], so that cultural attitudes toward the use of technology, affordability, and access can be a focus in their development. Inclusion and diversity within the development and testing of sensors are vital so that products are not biased and work equally regardless of skin color or other physiological differences [29]. Affordability is also a crucial point. Even though smartphone use is extensive [48] in both higher- and lower-income countries, the availability and quality of sensors differ across brands and models of smartphones. Wearable devices have far lower penetration in most high-income countries and lower still in those individuals with lower socioeconomic status. If accurate and reliable sensors are only available on high-end devices, then the net result will be inequitable outcomes. The consideration of a reverse innovation approach may be useful here if it is possible, to focus testing on inexpensive, easy-to-use products that can be rolled out at scale.

### Acceptability and Adherence

Crucial to the successful implementation of any innovation is early insight into the user perspective, including acceptability, uptake, and correct use by the intended target group [49-51]. Yet, this consultation is often omitted or occurs too late in mHealth implementation, resulting in user burden; technical issues; poor designs; and ultimately the lack of uptake, adherence, and impact of the technology [49,52-54]. Indeed, there is currently an absence of research on user perspectives on wearables and smartphone technologies for the detection of cancer. While key issues such as cost, motivation, comfort, ease of use, trust in data use, visibility, and interpretability of data are applicable across the spectrum of wearables and smartphone technologies in health [40,55], there may be additional, specific challenges for using these innovations for the detection of cancer.

In this context, acceptability also pertains to individuals’ willingness to share their data from smartphones and wearables with researchers, medical professionals, or private companies. Willingness to share data from wearables was reported to be lower than that for other commercial data [56]. Less than 15% (n=65) of survey respondents aged 60 years and older were willing to share wearable device data with academic research institutions and only 40% (n=423) of those aged 18-59 years were willing to do so. Trust in organizations and worry about data misuse have been shown to be a key factor in people’s willingness to share commercial data for health research [56]. Ensuring clear and transparent data use and data-sharing policies is vital for success. There are real concerns about the misuse of data, commercialization, and access to data by unauthorized people [57]. While data sharing is an essential component in the use of smartphones and wearables for cancer detection, data protection is equally as vital.

In research studies of wearables, dropout rates can be up to 44% [40], and nonadherence to wearing devices for the study duration can be up to 50% [58,59]. Nonadherence is likely to be even higher in people with preexisting comorbidities and for technologies requiring long-term engagement, as may be needed for cancer detection to track signals over time. Balancing the advantages and disadvantages of continuous versus intermittent measurements at certain intervals should be a key consideration.

It is, therefore, essential to investigate user perspectives in parallel with the development and potential future deployment of wearables and smartphone technologies for cancer detection. This also includes encompassing the views of clinicians who are involved in the ongoing surveillance and care of those with a history of cancer and would inevitably be involved in shared decision-making on the potential implementation of such technologies and, crucially, the ongoing clinical management of individuals whose sensors indicate signals of possible cancer. In general, primary care clinicians have not typically been deeply engaged in the implementation of other consumer-grade or regulated medical devices; understanding from these clinicians’ viewpoint on how they could use information from smartphones and wearables within their clinical care pathways is critical to any adoption [60].

### Unintended Consequences on Individuals and Health Care Services

The exciting potential of wearable technologies for cancer detection must be considered alongside the possible negative consequences. As seen with other new developments in cancer detection, given the overall very low prevalence of cancer, even tests with very high specificity will lead to a large number of individuals with false positives. The subsequent need for investigation and resources needed to differentiate those with false versus true positives (ie, do have cancer) could be considerable. For the majority of individuals, this could lead to huge risks of overinvestigation and inappropriate use of health care resources [61]. For cancer detection specifically, we can anticipate a far higher potential for wearables and smartphone technologies to generate anxiety than for other conditions (eg, detection of sleep apnea, or detection of irregular heartbeat), especially among those already fearful of cancer recurrence. This is particularly relevant to the question of how “results” should be delivered to users, what support would be needed at that time, and whose responsibility this would be. On the other hand, wearable use may lead to a false sense of reassurance, leading to a lower perceived need to attend cancer screening or respond to symptoms (eg, “my wearable says I am healthy...there is no need to see my doctor”). This is similar to when a negative cancer screening test result can overly reassure patients and affect subsequent decisions to seek care [62]. These issues about the psychological and behavioral impact of smartphone technologies and wearables to detect cancer remain unexplored and need focused behavioral science research.

## Conclusions

For most cancers, the time from detecting a bodily change to interpreting that change as requiring the advice of a health care professional constitutes a substantial proportion of the time prior to diagnosis. The detection of cancer remains one of the most prominent priorities of many health systems, governments, and private and public research funders [63,64], and “leaving no stone unturned” in technologies that could potentially improve early detection is a priority. The rapid advances in the hardware (ie, sensors) and software embedded in smartphones and wearables offer exciting and potentially untapped opportunities to detect early warning signs of cancer that may otherwise be missed. The research and development needed to advance this field include the selection of appropriate signals and development of effective sensors followed by robust clinical research into accuracy in real-world settings. This relies on the up-front specification of the target groups and their needs. Target product profiles should be developed specifically for cancer detection technologies, and innovators should consult these and consider regulatory challenges early in the process of development, to design products in line with the requirements of individuals, clinicians, and health care systems. The potential negative consequences of this type of technology should be acknowledged and investigated up-front, and mitigations should be incorporated into the design and implementation strategies. To avoid exacerbation of inequalities in cancer outcomes, research into the use of wearables and smartphone technologies in cancer detection should be done with an equity lens to ensure that products are developed for those who have poorer health outcomes, for whom new innovations could have the most impact. There is a need for research to explore the patient, public, and health care perspectives about the use of smartphones and wearables for the early detection of cancer while this field is in its infancy, so that these can be incorporated into product design to optimize acceptability and adherence, avoid unintended consequences, and maximize the chance of their success.

## Abbreviations

FDA: Food and Drug Administration
NICE: National Institute of Health and Care Excellence
NHS: National Health Service
mHealth: mobile health
